# Contemporary 0.55 T MRI for Lung Disease Assessment in Children and Young Adults with Cystic Fibrosis: A Proof-of-Concept Study

**DOI:** 10.3390/tomography12090126

**Published:** 2026-08-31

**Authors:** Nadine Bayerl, Maximilian Hinsen, Stephan Ellmann, Armin M. Nagel, Lisa C. Adams, Teresa Gerhalter, Rafael Heiss, Tobias Bäuerle, Sabina Schmitt-Grohé, Michael Uder, Oliver Rompel, Alexander Schnell

**Affiliations:** 1 Institute of Radiology, Uniklinikum Erlangen, Friedrich-Alexander-Universität Erlangen-Nürnberg, Maximiliansplatz 3, 91054 Erlangen, Germany; 2 RNZ—Radiologisch-Nuklearmedizinisches Zentrum, Martin-Richter-Str. 43, 90489 Nürnberg, Germany; 3 Department of Diagnostic and Interventional Radiology, Technical University of Munich, Ismaninger Str. 22, 81675 Munich, Germany; 4 Department of Neurology, Medical University of Graz, Auenbruggerplatz 22, 8036 Graz, Austria; 5 Department of Diagnostic and Interventional Radiology, University Medical Center of the Johannes Gutenberg University Mainz, Langenbeckstr. 1, 55131 Mainz, Germany; 6 Department of Pediatrics and Adolescent Medicine, Uniklinikum Erlangen, Friedrich-Alexander-Universität Erlangen-Nürnberg, Loschgestr. 15, 91054 Erlangen, Germany

**Keywords:** magnetic resonance imaging, cystic fibrosis, lung diseases, radiography, thoracic, respiratory insufficiency, diagnostic imaging, low-field MRI, 0.55 T, interobserver agreement

## Abstract

**Background**: Lung surveillance in cystic fibrosis (CF) relies on chest radiography (CR) despite cumulative radiation. Low-field MRI may be a radiation-free alternative for children and young adults. **Methods**: We prospectively compared 0.55 T MRI and CR in 28 same-day examinations of 22 people with CF (mean age 13 ± 5 years). Three raters scored the disease with the MRI CF score and a modified Chrispin–Norman score, expanded from two to three zones per hemithorax to match the six MRI lobes. Analyses used per-examination values averaged across the raters (Wilcoxon tests, intraclass correlation coefficients, ICC). **Results**: MRI yielded higher global scores (median 8.2 vs. 6.7, *p* = 0.002) and higher centrilobular opacity scores (*p* = 0.005, adjusted *p* = 0.03). Air trapping was also higher on MRI (*p* = 0.025) but not after correction for multiple comparisons (adjusted *p* = 0.12). The other categories did not differ. Both correlated inversely with FEV1 (*p* < 0.001), without a significant modality-by-FEV1 interaction (*p* = 0.08). Interobserver agreement was higher for MRI (ICC = 0.93) than CR (0.82), difference 0.12 (95% bootstrap CI 0.06 to 0.18). The findings held in a per-participant sensitivity analysis. **Conclusions**: 0.55 T MRI is a feasible, radiation-free alternative to CR in CF, yielding higher modality-specific scores and more consistent interobserver agreement. Because the instruments were only category-mapped and no CT reference was available, higher scores do not establish superior lesion detection.

## Simple Summary

Cystic fibrosis is an inherited disease that gradually damages the lungs, so patients need repeated imaging throughout their lives. Chest X-rays are widely used, but they involve radiation that accumulates over time, which is a particular concern for children and young adults. This study evaluated first experiences with a modern low-field MRI scanner as a radiation-free alternative. In 28 examinations of 22 young patients, MRI produced higher lung disease scores than chest X-rays and more consistent results between readers. Because the two methods were graded with different scoring systems and no CT scan was available for comparison, the higher MRI scores alone do not prove that MRI detects more disease. These findings suggest low-field MRI may represent a reliable monitoring option, encouraging larger studies.

## 1. Introduction

Cystic fibrosis (CF) is a life-limiting disease resulting from mutations within the cystic fibrosis transmembrane conductance regulator (CFTR) gene that encodes for a cAMP-regulated chloride channel [1]. Although CF affects multiple organ systems, end-stage lung disease remains a major cause of mortality according to the 2023 report of the Cystic Fibrosis Foundation [2]. In recent years, therapeutic advances such as highly effective CFTR modulators and structured monitoring programs, including regular lung function tests (LFTs) and pulmonary imaging, have contributed to a reduced incidence of advanced lung disease (ALD) and a continuous increase in overall median survival [2]. LFTs may be challenging in younger children because of limited cooperation, highlighting the need for alternative monitoring methods in this age group. While conventional chest radiographs (CR) are still performed at regular intervals for disease monitoring in many hospitals [3,4], the “iMAging managEment of cySTic fibROsis” (MAESTRO) consortium recommends CT or MRI as preferred imaging methods [4]. CT use is limited by ionizing radiation, particularly in pediatric patients. The dose of a single radiograph is far lower, but the burden accrues over a lifetime of surveillance. In a pediatric CF cohort of 77 children followed over 658 patient-years, chest radiographs accounted for 1485 of 1757 imaging examinations, and the mean cumulative effective dose reached 6.2 mSv (range 0.04–25) at a mean age of 9.5 years [5]. This corresponds to approximately 2.3 chest radiographs per patient-year in that cohort. Current consensus documents on CF lung imaging do not prescribe a fixed radiographic interval, so this frequency reflects institutional surveillance practice rather than a guideline-mandated schedule. In a larger mixed-age cohort, 16% of patients exceeded a cumulative effective dose of 20 mSv [6]. For a single posteroanterior chest radiograph, the German national diagnostic reference levels specify a dose-area product of 2.5 cGy·cm^2^ for children weighing 19 to under 32 kg, 4.0 cGy·cm^2^ for those weighing 32 to under 56 kg, and 12 cGy·cm^2^ for adults [7]. These levels represent the 75th percentile of the national distribution rather than optimized values. MRI offers comparable assessment of CF lung disease but remains restricted to tertiary centers due to availability and cost. Beyond these practical constraints, lung MRI at 1.5 T and 3 T is limited by the very short transverse relaxation times of lung parenchyma and by susceptibility gradients at the numerous air–tissue interfaces, which cause rapid signal decay and local distortion. Robust parenchymal imaging at these field strengths therefore often requires specialized ultrashort echo time techniques that are not universally available. In children, the narrower bore and higher acoustic noise of high-field systems additionally reduce tolerability and increase the need for sedation [8]. Recent technical breakthroughs have profoundly improved lung MRI imaging quality in terms of signal-to-noise ratio (SNR), resolution and contrast, including specialized sequences like proton density-weighted (PDw) with enhanced motion robustness [9]. Reduced manufacturing, transportation and operational costs make contemporary 0.55 T MRI systems more affordable compared to higher field-strengths [10,11,12]. The 0.55 T MRI is particularly advantageous for lung imaging as it reduces susceptibility artifacts in the lung parenchyma and at air–tissue interfaces, while longer transverse relaxation times enable lung imaging with conventional acquisition techniques [13,14]. Clinically, 0.55 T MRI has already shown its capability for detailed morphological lung imaging, e.g., in the visualization of inflammatory and fibrotic changes, nodules and cystic diseases such as lymphangioleiomyomatosis (LAM) [14,15,16,17]. Similar technical benefits are expected to apply to the visualization of CF lung changes, although studies specifically addressing CF-related lung imaging at contemporary 0.55 T are limited [18,19]. In this exploratory study, we aimed to address this gap and, with an initial series of patients, to investigate the feasibility of contemporary 0.55 T lung MRI for CF-related lung changes with special focus on the surveillance of children and young adults.

## 2. Materials and Methods

This single-center study was approved by the local ethics committee of the Friedrich-Alexander-Universität (FAU) Erlangen-Nürnberg (vote number 20-485-B) and was performed in accordance with the Declaration of Helsinki. Written informed consent was obtained from all the study participants prior to study inclusion and, for participants under 18 years of age, additionally from their parents or legal guardians.

### 2.1. Study Population

Between March 2022 and November 2023, a total of 40 people with CF (pwCF) attending the Center for Cystic Fibrosis of the Department of Child and Adolescent Medicine of the University Hospital Erlangen for routine annual imaging check-ups with conventional CR were offered an additional 0.55 T MRI examination. A total of 23 pwCF and their parents (or guardians, respectively) approved study participation, contributing 29 examinations. The patients received LFT and CR as standard care on the same day. For study participation, additional to the routine check-up including CR and LFT, an MRI of the lung was performed on the same day. One 9-year-old female patient canceled the MRI exam before its completion and was subsequently excluded from the study, leading to 28 of the 29 initiated examinations being completed (96.6%). The examination was discontinued at the patient’s request rather than because of a technical failure or a scheduling problem. No images of this examination entered the analysis, so the discontinuation does not affect the reported scores, and it is reflected in the completion rate given above. This left a total of 28 examinations that were included in the further analysis comprising CR, MRI and LFT performed on the same day. The 28 examinations were obtained in 22 pwCF. Six participants attended two consecutive annual check-ups during the recruitment period and therefore contributed two examinations each. No patients required sedation for the MRI examination.

### 2.2. Morphologic Lung Imaging

#### 2.2.1. Magnetic Resonance Imaging

Non-contrast enhanced MRI scans were acquired on a 0.55 T scanner (MAGNETOM Free.Max, Siemens Healthineers, Forchheim, Germany) with a bore size of 80 cm. The patients were positioned head-first supine with arms down. Phased-array receiver coils including a 6-channel flex coil, a 9-channel spine coil, and a 12-channel head and neck coil were used. The additional application of the head and neck coil provided a more homogeneous signal within the upper thoracic aperture. Two-dimensional turbo spin-echo (TSE) sequences were performed in transverse and coronal plane with free-breathing and gated image acquisition during expiration. In transversal plane, PDw sequences, and in coronal plane, T2-weighted (T2w) sequences with a short-tau inversion recovery (STIR) preparation were measured. Both sequences used a multi-shot turbo spin-echo readout along a radial k-space trajectory of rotating blades (BLADE, the vendor implementation of PROPELLER) rather than a half-Fourier single-shot readout. The transverse PDw sequence was acquired with an echo train length (turbo factor) of 13, a receiver bandwidth of 548 Hz/pixel and a BLADE coverage of 100%, and the coronal T2w STIR sequence with an echo train length of 23, a receiver bandwidth of 301 Hz/pixel and a BLADE coverage of 121.1%. The inversion time of the STIR preparation was 100 ms, shorter than the values customary at 1.5 T and consistent with the shorter T1 relaxation times at 0.55 T, since the inversion time nulls fat scales with the T1 of fat and is therefore shorter than at 1.5 T. The field of view was 380 mm for the transverse and 400 mm for the coronal acquisition, with 50% phase oversampling, one average, a slice thickness of 6.0 mm and an interslice gap of 1.2 mm. Respiratory gating was prospective and navigator-based, using a liver dome navigator with an acceptance window of 2.0 mm and an acquisition window covering 35% of the respiratory cycle. The liver dome was selected as the navigator position because the interface between the liver parenchyma and aerated lung yields the highest available signal contrast for edge detection, and because the craniocaudal excursion of the diaphragm is the dominant component of respiratory lung motion. The effective repetition interval, along with the acquisition time, followed the individual respiratory cycle so that the repetition times in Table 1 are the nominal protocol values. The DICOM headers of every scored series were reviewed to confirm that these settings applied throughout: echo train length (13 and 23), phase sampling (100%, excluding any half-Fourier acquisition), inversion time (100 ms), receiver bandwidth, slice thickness and interslice gap were identical in every examination. Across the cohort the realized repetition time was a median of 4114 ms (range 2778 to 7492) for the transverse PDw sequence and 4568 ms (range 2869 to 7755) for the coronal T2w STIR sequence, and the realized acquisition times had medians of 5:58 min (range 4:22 to 10:50) and 7:20 min (range 4:12 to 11:20), respectively. The spread reflects respiratory triggering. The transverse acquisition covered a median of 34 slices (range 28 to 44), and the coronal acquisition covered 25 slices (range 21 to 32). Examinations were acquired under vendor software versions syngo MR XA50, XA60 and XA80 with unchanged sequence parameters, and the header review reported above confirms that these parameters were identical across all three versions. A version-specific influence on image reconstruction cannot be excluded formally, but the raters graded morphological findings on a three-point ordinal scale rather than quantitative signal values, which makes the scores insensitive to the small differences in reconstruction that a software release may introduce. Table 1 provides detailed information of the acquisition parameters.

#### 2.2.2. Digital Chest Radiography

Digital CR was performed in erect posterior–anterior projection at full inspiration in all 28 examinations. The examinations were acquired with a combined fluoroscopy and radiography system (Luminos dRF Max, Siemens Healthineers GmbH, Forchheim, Germany) and a corresponding image post-processing engine (DiamondView MAX, Siemens Healthineers GmbH, Forchheim, Germany). The tube voltage setting was 80 kV in children 5–8 years old, 102 kV in children 8–15 years old, and 125 kV in adolescents >15 years and adults. These settings are those of the organ programs preconfigured on the radiography system for pediatric and adult chest examinations, and they were applied as in clinical routine rather than chosen for the study. The consistency of the resulting exposure across the cohort is documented by the median dose-area products reported below, which stayed well below the national diagnostic reference level in every weight class. For dose reduction, aluminum (1 mm) and copper (0.2 mm) filter systems were applied in all the patients. The dose-area product recorded for each radiograph was extracted from the DICOM header. The median values were 0.43 cGy·cm^2^ for participants weighing 19 to under 32 kg (*n* = 9), 0.67 cGy·cm^2^ for 32 to under 56 kg (*n* = 11) and 5.44 cGy·cm^2^ for 56 kg and above (*n* = 8), corresponding to 17%, 17% and 45% of the respective German national diagnostic reference level. One radiograph in the 32 to under 56 kg class reached 106% of its reference level. It was acquired in a 14-year-old weighing 55.3 kg using the adult protocol, 0.7 kg below the weight threshold of the adult reference category, against which it corresponds to 36%. This single value therefore reflects the reference category against which the examination is compared rather than an increased exposure, since the same radiograph corresponds to 36% of the reference level of the adult protocol that was applied.

### 2.3. Lung Function Tests

LFTs were performed on each patient with body plethysmography (Vyntus Body, Vyaire Medical, Hoechberg, Germany). Body plethysmography yielded FEV1, FEV1/FVC and total lung capacity for the entire cohort. The additional plethysmographic parameters were retained for the exploratory analysis reported in Section 3.2. The forced expiratory volume in one second (FEV1, percentage predicted as defined by the Global Lung Initiative Reference Values) was used for this study as a measure regarding the presence of an obstructive lung disorder [20].

### 2.4. Image Analysis

For MRI analysis of the pulmonary impairment, we applied the established “MRI CF score”, a semi-quantitative morphology scoring system for non-contrast enhanced MRI reporting the following findings [21]: (1) bronchiectasis with peribronchial wall thickening (per definition assessed as one MRI category due to the limited spatial resolution), (2) mucus plugging, (3) centrilobular opacities (nodules or tree-in-bud pattern), (4) sacculations, (5) consolidations and (6) air trapping. In MRI, the visibility of bronchiectasis is limited if there is no inflammatory thickening of the peribronchial walls. Therefore, the category “bronchiectasis with peribronchial wall thickening” was summarized consisting of two features [21,22].

For CR analysis, we used the semi-quantitative Chrispin–Norman scoring (CNS) system [23,24,25]. In CR, we reported the following features: bronchial line shadows (to represent bronchiectasis with bronchial wall thickening as a corresponding MRI category), ring shadows (as a corresponding feature to the MRI category sacculations), mottled shadows > 0.5 cm (as a corresponding feature to the MRI category mucus plugging), mottled shadows < 0.5 cm (as a corresponding feature to the MRI category centrilobular opacity/tree-in-bud pattern), overinflation (as a corresponding feature to the MRI category air trapping), and large soft shadows (as a corresponding feature to the MRI category consolidation and collapse).

The ratings were assessed in a semiquantitative way for each feature on a per-lobe basis on MRI (right upper lobe, right middle lobe, right lower lobe, left upper lobe, lingula, left lower lobe) and a per-zone basis on CR (right upper zone, right middle zone, right lower zone, left upper zone, left middle zone, left lower zone). For the regional assessment of the CR scoring, the CNS was slightly modified, as it originally only uses the upper and lower zone on each side instead of the upper, middle and lower zone, as applied by us. This three-zone modification was chosen to match the anatomical granularity of the six MRI lobes used for the paired per-region comparison and to allow finer cranio-caudal localization, at the cost of direct numerical comparability with two-zone Chrispin–Norman totals reported in earlier CF literature. The score has been modified before, both by omitting the lateral projection and in a reliability-tested regional variant, but the present three-zone version has not been separately validated, and its reliability is reported here for the first time. The grade definitions applied within each region were left unchanged, so the modification refines the spatial sampling of the instrument rather than its scoring rules, and the resulting interobserver agreement is reported for every category in Section 3.4 rather than assumed from the two-zone literature.

No study-specific scoring manual was written. The raters applied the published grade definitions of the MRI CF score [22] and of the modified Chrispin–Norman score [24] directly, and the illustrative examples of grades 0, 1 and 2 in the corresponding original publications served as an image atlas during reading. A Likert scale was utilized for quantifying the changes of each category: score of 0 (pathology not present), score of 1 (pathology present but not marked) and score of 2 (pathology clearly marked). The total score for each category was calculated as the global MRI CF score or global CR CF score, with a maximum possible score of 72 points, accounting for all 6 lobes (considering the lingula as a separate lobe). Each category could achieve a maximum score of 12 points. Scoring was performed by three independent radiologists with professional experience of 4/6/30 years (MH, NB, OR), respectively. Before the independent reading, all three raters attended a joint 15 min training session in which both scoring systems and the corresponding example images were reviewed. The session was intended to refresh the published grade definitions for readers already familiar with both instruments and not to calibrate reading thresholds on study cases. The consequences of this choice are addressed in the Limitations. For each rater and each scoring category, the transversal PDw and the coronal T2w sequences were reviewed in conjunction, and a single integrative score per lobe was assigned according to the predefined criteria. Scoring was thus performed per lobe on MRI, per zone on CR and per lesion category on the 0–2 Likert scale, and no per-patient composite or per-individual-lesion scoring was applied. Lesions extending across more than one lobe or zone were scored in each region they involved. The three raters scored all the examinations independently and were blinded to each other’s scores and to the clinical and lung-function data (including FEV1 and CFTR modulator status), and the MRI and the CR of a given patient were assessed in separate reading sessions.

### 2.5. Statistical Analysis

Prism version 10.2.0 (GraphPad Software, LLC, Boston, MA, USA) was used to perform Shapiro–Wilk tests and Wilcoxon signed-rank tests. The Shapiro–Wilk tests revealed significant deviations from normal distribution between the scoring groups. The values are therefore presented as median and interquartile range (IQR). For every examination and modality, the scores of the three raters were averaged into a single per-examination value. All the descriptive statistics, paired tests and regression analyses were then performed on these per-examination values so that 28 examinations, rather than pooled reader-level observations, entered these analyses. Nonparametric paired-group Wilcoxon signed-rank tests evaluated the differences between the MRI and CR groups. RStudio version 2022.12.0 + 353 (Posit Software, PBC, Boston, MA, USA) and the R package irr were used to calculate the intraclass correlation coefficient (ICC) for interobserver agreement. A two-way mixed-effects model with absolute agreement and single-rater unit was applied (according to Koo and Li [26]), as the three radiologists represented the only raters of interest rather than a random sample from a larger pool of potential raters. ICCs are reported as point estimates with 95% confidence intervals (CI). The global ICC was calculated by pooling all the category × lobe-level ratings across examinations (*n* = 1008 observations per rater). This item-level pooling preserves the full inter-rater variability and is consistent with the category-specific ICCs. Aggregating instead to a single per-examination sum score was deliberately avoided, as summation averages out inter-rater variability while retaining the large between-examination range (0–72) and thereby inflates the ICC. ICC was preferred over kappa because the quadratically weighted kappa and the ICC are equivalent reliability coefficients for ordinal ratings [27], because the ICC is natively defined for three raters, whereas Cohen’s kappa is limited to two, and because it yields coefficients on a common scale at the item and score levels. Because the pooled global ICC treats the 36 item-level ratings of an examination as independent observations, its confidence interval is narrower than the effective sample size of 22 participants would justify and is reported descriptively. As a sensitivity analysis, ICCs were recomputed on per-examination sum scores. The difference between the global MRI and CR ICCs was quantified by a bootstrap with the participant as the resampling unit (5000 replicates), which respects both the nesting of items within an examination and the repeated examinations of six participants. ICC values < 0.5 were considered as poor agreement, values between 0.5 and 0.75 as moderate agreement, values between 0.75 and 0.9 as good agreement, and values > 0.90 as excellent agreement [26]. Because the MRI CF score and the Chrispin–Norman score are distinct instruments rather than repeated measurements of the same quantity, a Bland–Altman plot of the difference between the global MRI and CR scores against their mean was generated in R version 4.6.0 (R Foundation for Statistical Computing, Vienna, Austria) to descriptively visualize the direction and magnitude of the systematic difference between the two modalities across the range of disease severity, rather than as a formal test of interchangeability. Limits of agreement and the slope of the difference against the mean are therefore reported as descriptive effect sizes without inferential interpretation. Because the mean of two scores carries the measurement error of both, the question of whether the gap between modalities widens with disease burden was instead addressed against the external severity axis FEV1, using the modality-by-FEV1 interaction term specified below. Correlation between LFT and global lung score was assessed by linear regression analysis using Prism 10.2.0. Additional analyses were performed in Python 3.14 with NumPy 2.4, SciPy 1.17 and statsmodels 0.14, namely, the linear mixed-effects model of the modality-by-FEV1 interaction, the bootstrap of the difference between the two intraclass correlation coefficients, the leave-one-rater-out analyses, the sensitivity analysis restricted to one examination per participant, the Holm–Bonferroni correction, the category-level *p*-values and the exploratory regressions on FEV1/FVC and total lung capacity. Figures were drawn in R. For each analysis, the slope co-efficient, as well as the 95% confidence interval are indicated. Rather than comparing the two slopes by inspecting the overlap of their confidence intervals, the difference between them was tested formally in a combined linear mixed-effects model of score on FEV1, modality and their interaction, with the patient as a random effect. For *p*-values < 0.05 statistically significant differences, and for *p*-values < 0.01 highly statistically significant differences were assumed. To account for the six category-level comparisons, category *p*-values were adjusted for multiple testing using the Holm–Bonferroni method. The global comparison was the primary endpoint and was not part of this correction. Because the two instruments are not interchangeable measures of the same quantity, this comparison quantifies the difference in severity assigned under the pre-defined category mapping between the MRI CF score and the modified Chrispin–Norman score. It does not establish which modality is closer to the true extent of disease. That both scales reach a maximum of 72 points is a consequence of their construction, six categories by six regions by a maximum of two points, and not evidence of a common metric. This study is reported in accordance with the Guidelines for Reporting Reliability and Agreement Studies (GRRAS) [28].

## 3. Results

### 3.1. Study Population

A total of 28 examinations in 22 pwCF with a mean age of 13 ± 5 years at examination, ranging from 6 to 27 years, including 11 examinations in female and 17 in male participants, included both 0.55 T MRI of the lung and CR on the same day. Refer to Table 2 for detailed patient metrics including age, mutation status, and FEV1.

### 3.2. Correlation with Lung Function Tests

**Figure 1:**
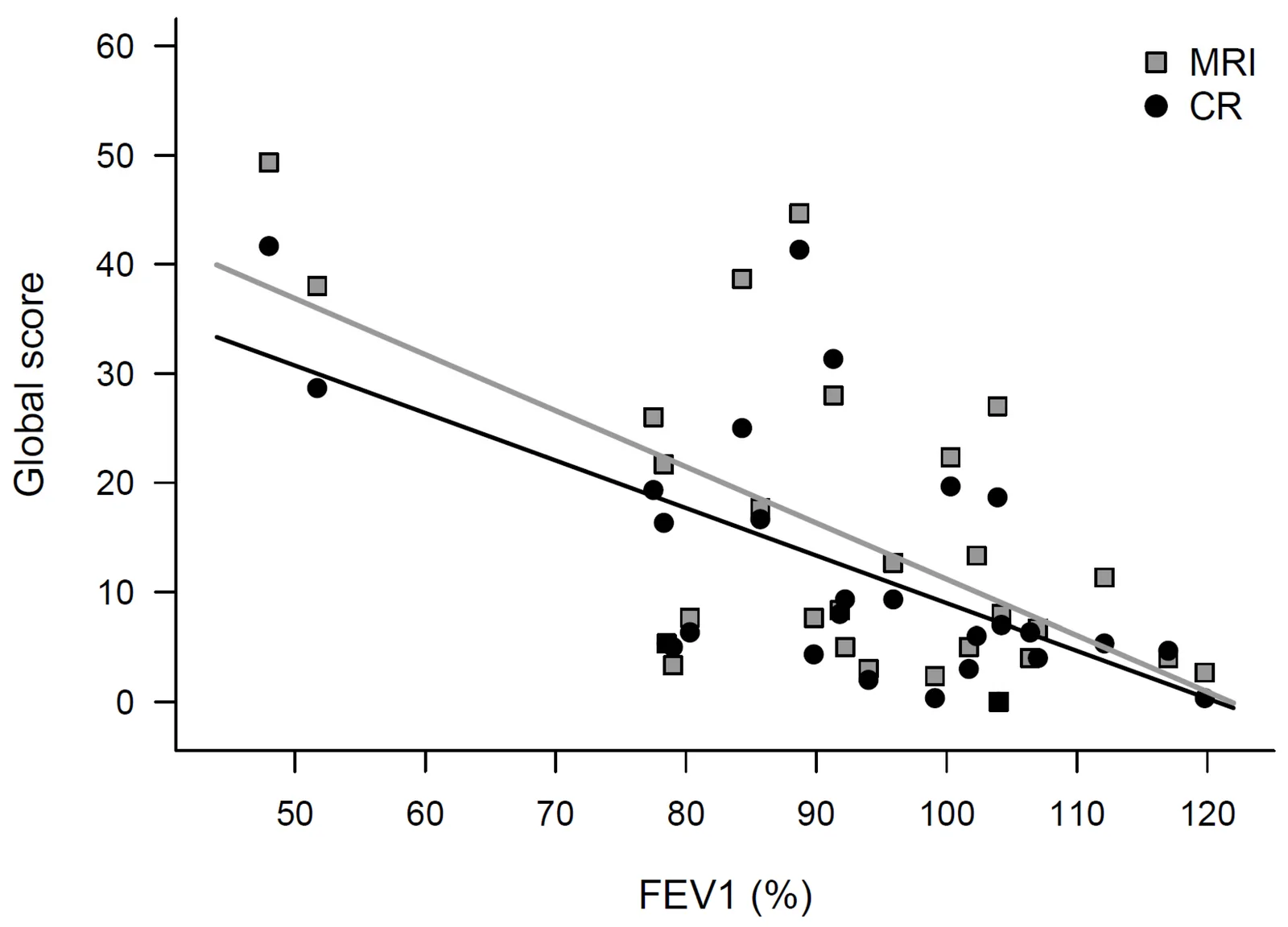
Regression analysis of lung function (FEV1) and per-examination global scores, each averaged across the three raters, showing higher MRI than chest radiography scores across the range of lung function, while both were significantly inversely correlated with FEV1. The two slopes did not differ significantly (interaction *p* = 0.08). Grey squares and the grey line denote MRI, black circles and the black line chest radiography; the lines are the corresponding linear regressions. FEV1, forced expiratory volume in one second, percentage predicted.

Correlations with FEV1 as an objective measure for pulmonary function showed higher global scores on MRI than on CR across the range of lung function, whereas both modalities exhibited a significantly inverse correlation to FEV1 (each *p* < 0.001 slope MRI −0.51 [CI −0.77 to −0.25]; slope CR −0.43 [−0.65 to −0.21]), as depicted in Figure 1. In a combined mixed-effects model, the modality-by-FEV1 interaction term was not significant (beta = −0.079; 95% CI −0.168 to 0.010; *p* = 0.08), so the two slopes did not differ significantly. At this sample size this constitutes absence of evidence for a difference rather than evidence of equivalence. Only 6 of the 28 examinations had an FEV1 below 80% predicted and two below 60%, so observations at the severe end of the range rest on a small subgroup. In an exploratory analysis of the remaining plethysmographic parameters, both modalities correlated inversely with FEV1/FVC (MRI −0.52, 95% CI −0.91 to −0.14, *p* = 0.010; CR −0.40, 95% CI −0.73 to −0.06, *p* = 0.024). Correlations with total lung capacity were positive but did not reach significance (MRI 0.42, *p* = 0.09; CR 0.37, *p* = 0.07). FEV1/FVC is strongly correlated with FEV1 in this cohort (r = 0.81) and therefore does not constitute independent confirmation, whereas total lung capacity is largely independent of FEV1 (r = −0.15). These exploratory analyses were not corrected for multiplicity.

### 3.3. Image Analysis

**Figure 2:**
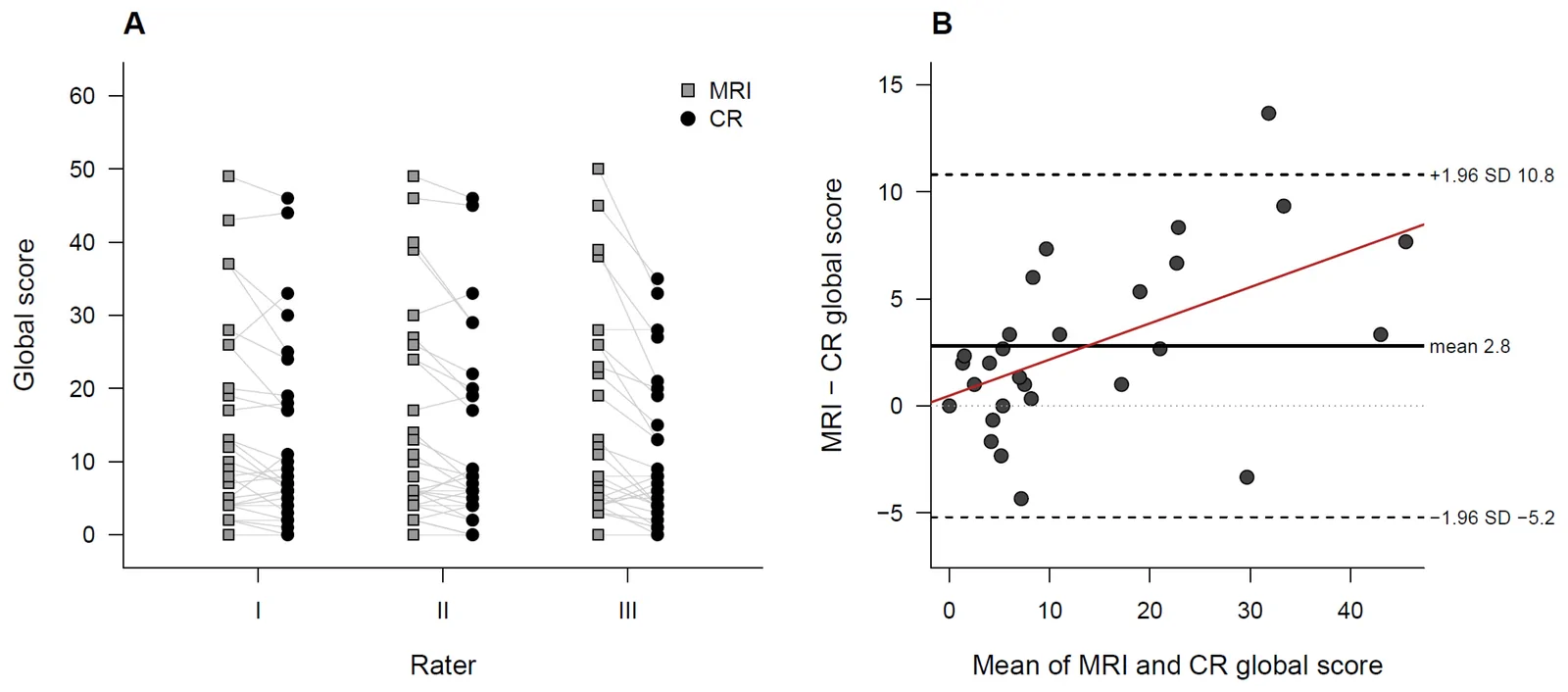
(**A**): Matched paired plots of the global MRI and chest radiography (CR) scores of all 28 examinations, shown separately for each of the three raters (I–III) to display the full reader-level data. Statistical testing was not performed on these reader-level observations but on the per-examination values averaged across the three raters (*n* = 28; see Section 2.5), which yielded significantly higher scores on MRI than on CR. (**B**): Bland–Altman plot of the difference between the global MRI and CR scores plotted against their mean, showing that MRI scores were systematically higher than CR (mean difference 2.8 points; 95% limits of agreement −5.2 to +10.8). Because the two scores are not interchangeable measures of the same quantity, the plot and the slope of the difference against the mean (0.169; 95% CI 0.061 to 0.277) are reported descriptively. In (**B**), the solid horizontal line marks the mean difference, the dashed lines the 95% limits of agreement, the dotted line zero difference, and the red line the linear regression of the difference on the mean.

The 0.55 T MRI yielded significantly higher scores in assessing global pulmonary manifestations (median = 8.17, interquartile range/IQR = 4.25–25.08) compared to CR (median = 6.67, IQR = 4.42–19.17; *p* = 0.002; Figure 2A). This difference reflects the severity assigned under the pre-defined category mapping between the two instruments and not a validated difference in lesion detection.

After Holm–Bonferroni correction for the six category-level comparisons, MRI scored significantly higher than CR for centrilobular opacities (mottled shadows < 0.5 cm on CR) (*p* = 0.005; adjusted *p* = 0.03; MRI, median = 1.83, IQR = 0–3.58; CR, median = 0.67, IQR = 0–3.00). MRI also scored higher than CR for air trapping (overinflation on CR) (*p* = 0.025; MRI, median = 2.83, IQR = 0–9.67; CR, median = 1.33, IQR = 0–6.25), but this difference did not remain significant after correction (adjusted *p* = 0.12). No significant differences were observed in assessing mucus plugging (*p* = 0.65), bronchiectasis including bronchial wall thickening (*p* = 0.22), consolidations (*p* = 0.07), or sacculations (*p* = 0.68) on MRI compared to CR. All the corresponding Holm–Bonferroni adjusted *p*-values were 0.28 or higher. In a leave-one-rater-out analysis, the global difference between the modalities was robust: it remained significant when any single rater was excluded (*p* = 0.0008, 0.002 and 0.009) and when each rater was analyzed alone (*p* = 0.03, 0.006 and 0.0005). The category-level difference in centrilobular opacities was not robust in the same way. It was no longer significant when the least experienced rater was excluded (*p* = 0.38) and was significant for that rater alone (*p* = 0.0006), which reflects the threshold difference for this feature reported in Section 3.4. For the detailed metrics refer to Table 3.

**Figure 3:**
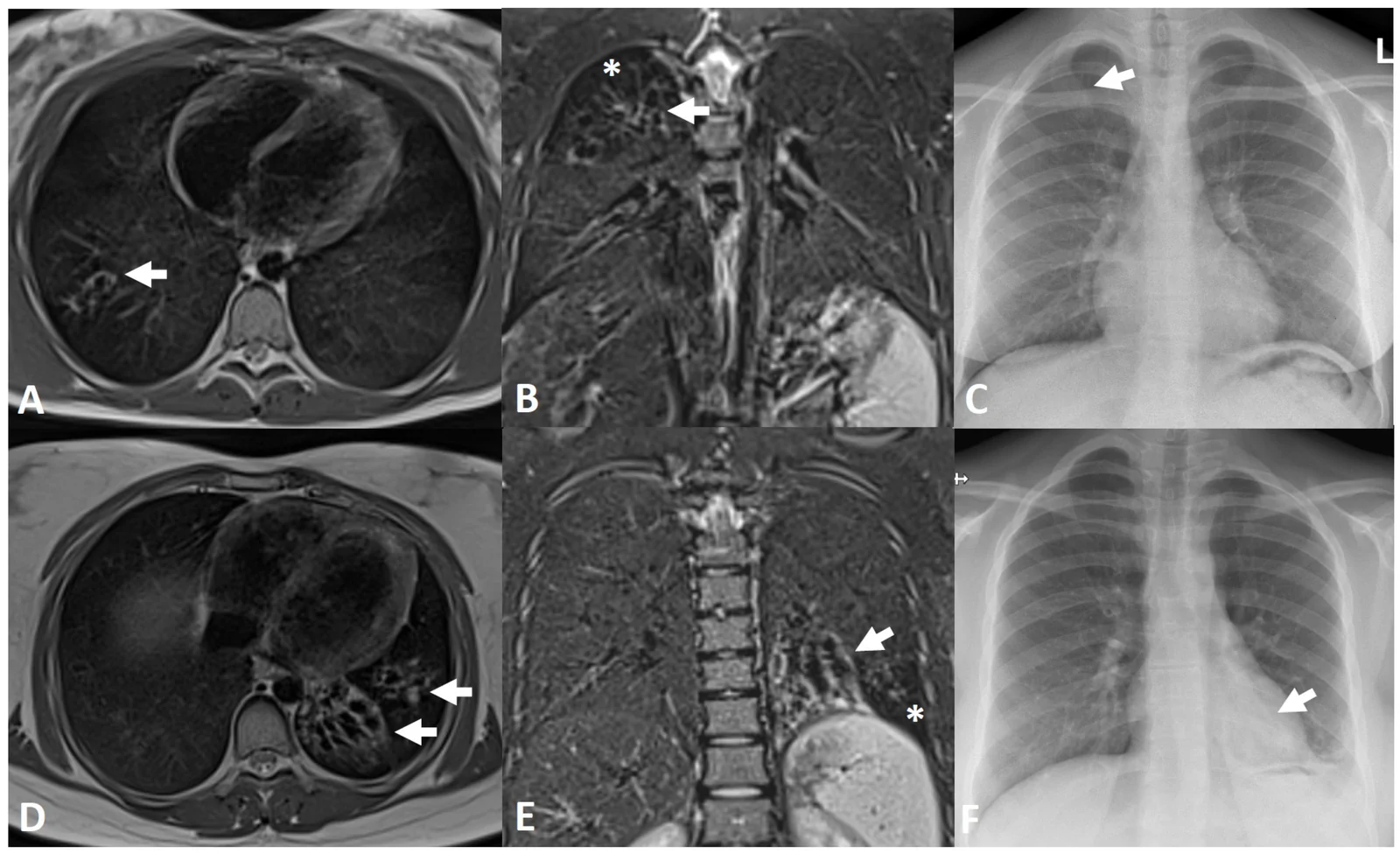
(**A**,**B**): MRI of the lung at 0.55 T in a female 14-year-old patient with CF presenting with bronchiectasis and air trapping in the right upper lobe (arrow), as well as air trapping (*) in the lingula. (**A**): Transversal proton-density-weighted sequence of the lung. (**B**): Coronal T2-weighted fat-suppressed sequence of the lung. (**C**): Corresponding chest radiography in posterior–anterior view revealing subtle or barely discernible pulmonary abnormalities, such as bronchiectasis in the right upper zone (arrow). (**D**,**E**): MRI of the lung at 0.55 T in a female 17-year-old patient with CF presenting with bronchiectasis (arrow) in the left lower lobe and air trapping (*) in the lingula. (**D**): Transversal proton-density-weighted sequence of the lung. (**E**): Coronal T2-weighted fat-suppressed sequence of the lung. (**F**): Corresponding chest radiography in posterior–anterior view not revealing bronchiectasis in the left lower lobe, as it is obscured by the cardiac silhouette (arrow). Blunted left lateral recess. L, left side marker on the radiograph.

The Bland–Altman plot of the difference between the global MRI and CR scores against their mean (Figure 2B) shows a systematic difference rather than agreement: MRI scores were, on average, higher than CR, and the difference increased with the mean of the two scores (slope 0.169; 95% CI 0.061 to 0.277). As the two instruments are not interchangeable measures of the same quantity, this plot is reported descriptively, and the slope is given as an effect size rather than as a test of proportional bias. The corresponding question of whether the gap widens with disease burden was tested against FEV1 and was not significant (*p* = 0.08, see above). Because six participants contributed two examinations each, all the analyses were repeated on one examination per participant. Restricting the analysis to the first examination of each participant (*n* = 22) yielded a median global score of 10.50 for MRI and 8.67 for CR (Wilcoxon *p* = 0.005), regression slopes against FEV1 of −0.59 for MRI and −0.49 for CR, and interobserver agreement of ICC 0.94 for MRI and 0.80 for CR with a difference of 0.144 (95% bootstrap CI 0.096 to 0.203, resampling participants rather than examinations). Restricting the analysis to the last examination of each participant gave concordant results (Wilcoxon *p* = 0.0003; ICC difference 0.129, 95% bootstrap CI 0.072 to 0.187). No conclusion of this study depends on whether the repeated examinations are retained or removed. For clinical showcases refer to Figure 3.

### 3.4. Interobserver Agreements

For MRI, interobserver agreement was good to excellent across all six scoring categories, with ICCs ranging from 0.86 (95% CI: 0.82–0.89) for bronchiectasis to 0.98 (95% CI: 0.97–0.98) for consolidation. For CR, interobserver agreement was good for the categories bronchiectasis (ICC = 0.84; 95% CI: 0.80–0.88), sacculation (ICC = 0.86; 95% CI: 0.82–0.89) and overinflation (as a corresponding CR category to the MRI category air trapping; ICC = 0.85; 95% CI: 0.81–0.89). The category mottled shadows > 0.5 cm (corresponding to mucus plugging on MRI; ICC = 0.83; 95% CI: 0.79–0.87) also showed good agreement, whereas large soft shadows (corresponding to consolidation/collapse on MRI; ICC = 0.72; 95% CI: 0.65–0.77) showed moderate agreement. Poor agreement between the three raters was observed for mottled shadows < 0.5 cm (as a corresponding CR category to the MRI category centrilobular opacity; ICC = 0.36; 95% CI: 0.23–0.48). Considering the six categories for MRI vs. CR, global interobserver agreement was excellent for MRI scorings (ICC = 0.93; 95% CI: 0.93–0.94) and good for CR scorings (ICC = 0.82; 95% CI: 0.80–0.83). The difference of 0.117 was confirmed by a bootstrap with the participant as the resampling unit (95% CI 0.062 to 0.183, no replicate of 5000 reached zero). In the sensitivity analysis on per-examination sum scores, both coefficients rose (MRI 0.99, CR 0.95), which illustrates that aggregation averages out inter-rater variability and inflates the ICC, and supports the item-level pooling used here. Collapsing the three CR zones per hemithorax back to the two zones of the original Chrispin–Norman score left the coefficient essentially unchanged, at an ICC of 0.82 under either of the two plausible collapsing rules against 0.82 for the three-zone grid, so the zone modification does not account for the reproducibility reported here. The poor agreement for mottled shadows < 0.5 cm on CR was systematic rather than random: agreement between the two more experienced raters was good (pairwise ICC 0.84), whereas the least experienced rater recorded this feature almost never (mean score 0.03 versus 0.36 and 0.29), reducing both pairwise coefficients involving that rater to near zero (0.005 and 0.021). The higher MRI coefficient does not merely reflect a wider score range: between-examination standard deviations differed by a factor of 1.18 (14.05 versus 11.90 on the sum score), whereas the mean absolute difference between raters differed by a factor of 1.9 (1.24 versus 2.38 points). The advantage therefore arises from smaller measurement error, not from a wider scale. Refer to Table 3 for the detailed ICC values of the MRI and CR scorings.

## 4. Discussion

This exploratory application study demonstrates that contemporary 0.55 T MRI provides a feasible radiation-free option for assessing pulmonary manifestations in pwCF. We found higher morphological scores and more consistent interobserver reproducibility with MRI than with CR, which is still widely used in many hospitals for disease monitoring, although diagnostic accuracy against an independent reference standard was not assessed. The higher MRI scores quantify the severity assigned under the pre-defined category mapping between two different instruments. Without a reference standard they do not establish that MRI detects more true lesions. Clinically, this means that MRI and CR scores are not interchangeable numbers and that a change of modality during surveillance cannot be read as disease progression. The good-to-excellent interobserver agreement for MRI (ICC 0.86–0.98) compared to more variable agreement for CR (ICC 0.36–0.86) suggests more reliable and consistent assessment of CF lung disease on 0.55 T MRI. This improved consistency in interpretation could lead to more standardized treatment decisions and better disease monitoring, all without the need for radiation exposure. Several factors likely explain why MRI yielded higher scores than CR. As a cross-sectional tomographic technique, MRI avoids the antero-posterior summation of overlapping structures that limits projectional radiography, and its higher soft-tissue contrast may allow a more direct depiction of mucus impaction and peribronchial inflammatory change, which produce only nonspecific increased density on CR. Without a reference standard, this remains a mechanistic explanation for the difference in score rather than a demonstrated gain in lesion detection. Air trapping is detected on MRI through regional differences in parenchymal signal and lobar volume on expiration-gated acquisitions, whereas CR relies on the comparatively insensitive surrogate of gross hyperinflation.

These results extend previous research and address key limitations of conventional imaging approaches. While 1.5 T and 3 T MRI systems have shown promise in CF imaging [21,22,29,30,31], they face limitations including cost, availability, and patient comfort, particularly for the pediatric population. The technical basis for the enhanced visualization of lung morphology in 0.55 T lung MRI lies in the fundamentally different tissue properties at lower field strength [14,32]. The longer T2* values in lung parenchyma at 0.55 T (T2* = 8.2 ms vs. 2.11 ms at 1.5 T and 0.74 ms at 3 T) enable robust imaging using conventional sequences, tackling a major limitation of 1.5 T and 3 T MRI [32,33]. Additionally, T2 values of lung parenchyma at 0.55 T have been quantified at approximately 60 ms [14,34], comparable to or slightly longer than at 1.5 T, which supports robust signal retention over the echo trains of the multi-shot BLADE readouts used in this study. In combination with the shorter T1 at 0.55 T [14,35] and the reduced susceptibility gradients at air–tissue interfaces inherent to lower field strengths, these relaxation properties enable robust morphological lung imaging with clinically established sequences without requiring specialized ultrashort echo time techniques [33], tackling a major limitation of higher field strengths. Direct comparison with the 1.5 T and 3 T CF-MRI literature is limited by heterogeneous scoring systems but is informative. Using morpho-functional MRI-CF scores, Eichinger et al. reported global-score concordance correlation coefficients of 0.94 to 0.98 [22], and Sileo et al. reported an inter-observer ICC of 0.84 for the same score, against 0.96 for HRCT in the same cohort [36]. Our 0.55 T MRI global ICC of 0.93, computed at the item-level, lies within this higher-field range (the corresponding value on aggregated sum scores is 0.99), suggesting that the longer T2* at a low field offsets the expected loss in spatial resolution rather than compromising reproducibility. Reproducibility is, however, not diagnostic accuracy: studies comparing MRI directly with CT found markedly lower MRI–CT concordance for bronchiectasis and air trapping (ICC 0.41 and 0.35, respectively) and MRI sensitivity for severe bronchiectasis as low as 0.33 [37,38]. As no CT was acquired in the present study, our results show only that 0.55 T MRI is at least as reproducible as CR in this surveillance setting, not that it can substitute for CT-based structural staging [39].

In our study, the significant inverse correlation between FEV1 values in both imaging modalities (*p* < 0.001) is consistent with their clinical utility, although this cross-sectional association is partly confounded by age and, in the modulator era, by the decoupling of preserved FEV1 from persistent structural change. MRI showed a numerically steeper correlation slope (−0.51 vs. −0.43), but the modality-by-FEV1 interaction term was not significant (*p* = 0.08), so this difference is not statistically established and may reflect differences in score dispersion rather than superior detection. Ciet et al. have emphasized the importance of sensitive imaging biomarkers for early disease detection [4]. In addition, the interobserver agreement in our study was good to excellent for MRI (ICC 0.86–0.98), exceeding the poorer interobserver agreement reported in our own CR results and in previous CR-based studies [24]. This finding suggests that low-field MRI may provide a reproducible assessment of CF manifestation. The FEV1 correlation was moreover based on the global composite score. Categories reflecting active airway obstruction, such as air trapping and mucus plugging, would be expected to track FEV1 more closely than categories reflecting fixed structural damage, such as sacculations or consolidation, and category-specific correlation with lung function should be examined in larger cohorts. A CFTR modulator was taken at 22 of the 28 examinations (13 elexacaftor–tezacaftor–ivacaftor, 5 ivacaftor, 2 lumacaftor–ivacaftor, 2 tezacaftor–ivacaftor) and at none of the remaining 6. The global scores and FEV1 did not differ detectably between these groups (MRI median 8.17 versus 9.33, *p* = 0.87; CR 7.50 versus 6.17, *p* = 0.91; FEV1 93.1 versus 95.5% predicted, *p* = 0.68), but with only six examinations in the untreated group, this comparison has no meaningful power and is reported only to document that modulator status does not visibly drive the observed score difference.

The clinical implications of our findings, if confirmed, could be considerable. The radiation-free nature of 0.55 T MRI addresses a critical concern in CF care, where patients require frequent imaging throughout their lives [40]. The larger 80 cm bore diameter of the MRI scanner used in this study, together with reduced operational costs, makes this technology potentially more accessible for pediatric centers and smaller non-tertiary care hospitals. Whether these economic advantages translate into equivalent diagnostic performance and reader expertise outside specialized centers has not been formally studied and should not be assumed [10,11,32,41]. The high interobserver agreement suggests more consistent disease assessment, potentially leading to more standardized treatment decisions. Recent recommendations from the MAESTRO consortium emphasize the importance of early detection of structural changes and disease progression, especially in young patients with disease-modifying potential [4]. In the era of highly effective CFTR modulators, which can exceed treatment costs of $100,000 per year, precise monitoring of treatment response becomes increasingly important [42]. Our study suggests that 0.55 T MRI could contribute to this capability, given its more consistent interobserver scoring of inflammatory and structural alterations. While some specialized tertiary centers in Germany have already adopted MRI as the standard of care [29], our findings with contemporary 0.55 T technology could help expand its adoption.

Several limitations warrant discussion. This is a monocentric proof-of-concept study, and its small sample of 28 examinations in 22 pwCF necessitates cautious interpretation. It was designed to establish feasibility and to generate effect-size estimates rather than to provide adequately powered confirmatory comparisons, and the findings require validation in larger, multicenter cohorts before clinical implementation. Although the three readers scored independently and were blinded to clinical data and to each other, all practice at the same institution and were familiar with the study aims and both scoring systems. This may introduce a shared expectation bias that independent scoring alone does not eliminate, particularly for features with softer visual thresholds such as centrilobular opacities, and independent replication by readers external to the study institution is therefore needed. The systematic divergence of one rater for mottled shadows < 0.5 cm on CR indicates that the reading thresholds for this feature were not aligned across the raters, which the brief joint training session did not prevent. Future studies should include a formal calibration on a shared training set with feedback on discordant cases and report reader-level agreement alongside the pooled coefficient. Because this feature also carries the only category-level difference that survived correction for multiplicity, and because that difference disappears when the diverging rater is excluded, the category-level result should be regarded as provisional until it is reproduced with calibrated readers. The global difference between the modalities does not depend on any single rater. Statistically, ICCs were computed on lobe-level observations, treating lobes as independent units (pseudoreplication). While consistent with prior radiology reproducibility studies, the pooled coefficient does not model the nesting of items within an examination. A linear mixed-effects model with the participant as a random effect was used for the comparison of the FEV1 slopes, whereas a variance-component model of the difference between two intraclass correlation coefficients is unstable at 22 clusters, so the bootstrap at the participant level and the sensitivity analysis on the sum scores were used for that comparison instead. Furthermore, categories with low pathology prevalence (median = 0 for mucus plugging, consolidation, and sacculation) may yield ICCs that partly reflect agreement on the absence of disease rather than on severity grading. Because many lobes were scored zero, this caveat applies equally to the pooled global ICCs. Six of the 22 participants contributed two annual examinations each, so the 28 examinations are not statistically independent. All the analyses were therefore repeated on one examination per participant, which left every conclusion unchanged (Section 3.3). The correlation with lung function was based primarily on FEV1. The parameters separating obstructive from restrictive components, FEV1/FVC and total lung capacity, were available from body plethysmography and are reported in Section 3.2 but only as an exploratory analysis without correction for multiplicity, and FEV1/FVC is itself closely correlated with FEV1 in this cohort. Finally, motion-related sequence repetitions, non-diagnostic acquisitions and structured patient-reported tolerability were not recorded systematically during the study, so these aspects of feasibility can be reported only as the completion rate. Additionally, CT, the established structural reference standard for CF lung disease, was not acquired. This study therefore reports differences in morphological score and interobserver reliability between MRI and CR, not diagnostic accuracy against an anatomical gold standard. CR was chosen as the comparator because it reflects our institution’s established surveillance practice in clinically stable pediatric and young adult patients and because the clinical question was whether MRI can replace the radiograph these patients already receive, not whether it matches CT. Adding a CT arm would have exposed a radiation-averse pediatric cohort to ionizing radiation, which would have contradicted the radiation-reduction rationale of the study. Because the CR grid was expanded from two to three zones per hemithorax to match the six-lobe MRI grid, absolute CR scores in this cohort are moreover not directly comparable with two-zone Chrispin–Norman totals reported in earlier CF literature. While the 0.55 T system already benefits from reduced susceptibility artifacts owing to its lower field strength, further optimization of sequence parameters and standardization of protocols across centers will be crucial for widespread adoption. To our knowledge, this is the first study evaluating a CF cohort using contemporary 0.55 T MRI, and further research is needed to benchmark its performance against 1.5 T and 3 T systems that have been extensively studied in CF imaging [21,22,29,30,43].

## 5. Conclusions

Our first-experience findings indicate that contemporary 0.55 T MRI provides a feasible, radiation-free approach to morphological lung assessment in children and young adults with CF, yielding higher modality-specific scores and more consistent interobserver scoring than chest radiography. Because the two modalities were assessed with distinct, only category-mapped instruments and no CT reference standard was available, the higher MRI scores cannot be attributed to superior true-lesion detection on the basis of these data alone. Whether this reproducibility advantage translates into diagnostic accuracy against CT, and whether it generalizes beyond a single-center cohort of 22 patients, requires confirmation in larger, multicenter studies with external readers.

## Figures and Tables

**Table 1:** Magnetic resonance imaging parameters.

	Acquisition Parameters	
**Sequence**	PDw TSE	T2w TSE STIR
**Orientation**	transversal	coronal
**TA median (range)**	5:58 min (4:22–10:50)	7:20 min (4:12–11:20)
**TE/TR**	35/2000 ms	77/2500 ms
**Slice thickness**	6 mm	6 mm
**In-plane resolution**	1.3 × 1.3 mm^2^	1.5 × 1.5 mm^2^
**Matrix**	304 × 304	272 × 272
**Acceleration mode**	GRAPPA	GRAPPA
**Acceleration factor**	2	2
**Readout trajectory**	PROPELLER (BLADE), ETL 13	PROPELLER (BLADE), ETL 23

PDw, proton density weighted; T2w, T2-weighted; TSE, turbo spin-echo; BLADE, vendor implementation of the PROPELLER trajectory; ETL, echo train length; STIR, short-tau inversion recovery; TA, time of acquisition; TE, time of echo; TR, time of repetition; GRAPPA, generalized autocalibrating partial parallel acquisition; PROPELLER, periodically rotated overlapping parallel lines with enhanced reconstructions.

**Table 2:** Study population with mutation status, LFT results and CFTR modulator treatment.

Examination ID	Age (Years)	Mutation 1	Mutation 2	FEV1 (%)	CFTR Modulator
1	7	G551D	Duplication Exon 7–11	104.0	I
2	27	F508del	F508del	48.0	ETI
3	10	F508del	F508del	92.2	LI
4	12	F508del	F508del	104.2	ETI
5	14	F508del	F508del	102.3	None
6	6	F508del	F508del	80.3	LI
7	7	F508del	F508del	79.0	ETI
8	10	F508del	G551D	89.8	I
9	11	F508del	G551D	94.0	ETI
10	12	F508del	G542X	91.8	ETI
11	19	F508del	G551D	103.9	I
12	14	F508del	F508del	78.3	ETI
13	9	F508del	R347P	106.4	None
14	10	F508del	R347P	117.0	ETI
15	9	F508del	D1152	78.5	None
16	17	F508del	D1152	77.5	ETI
17	12	F508del	F508del	85.7	ETI
18	18	F508del	F508del	99.1	ETI
19	17	F508del	F508del	88.7	None
20	17	G551D	Q39X	107.0	I
21	21	F508del	F508del	51.7	ETI
22	11	F508del	G551D	101.7	I
23	10	F508del	F508del	119.8	None
24	7	F508del	F508del	91.3	TI
25	8	F508del	F508del	100.3	ETI
26	19	F508del	F508del	95.9	TI
27	11	G542X	F861fs	84.3	None
28	15	F508del	F508del	112.1	ETI

I = Ivacaftor, L = Lumacaftor, T = Tezacaftor, E = Elexacaftor.

**Table 3:** Scoring metrics.

Scoring Categories	MRI-Median	MRI-IQR	MRI-ICC (95% CI)	CR-Median	CR-IQR	CR-ICC (95% CI)
Global	8.17	4.25–25.08	0.93 (0.93–0.94)	6.67	4.42–19.17	0.82 (0.80–0.83)
Bronchiectasis	4.33	2.75–6.58	0.86 (0.82–0.89)	4.33	3.00–6.67	0.84 (0.80–0.88)
Mucus plugging	0.00	0.00–1.50	0.96 (0.94–0.97)	0.167	0–1.58	0.83 (0.79–0.87)
Centrilobular opacity/mottled shadows < 0.5 cm	1.83	0–3.58	0.91 (0.89–0.93)	0.67	0–3.0	0.36 (0.23–0.48)
Air trapping/overinflation	2.83	0–9.67	0.93 (0.91–0.95)	1.33	0–6.25	0.85 (0.81–0.89)
Consolidation	0.00	0–0	0.98 (0.97–0.98)	0.00	0–0	0.72 (0.65–0.77)
Sacculation	0.00	0–3.33	0.94 (0.93–0.96)	0.00	0–1.92	0.86 (0.82–0.89)

Metrics of MRI and CR scoring including the categories’ global extent (sum of all other categories), bronchiectasis, mucus plugging, centrilobular opacity/mottled shadows < 0.5 cm, air trapping/overinflation, consolidation and sacculation. The metrics are given as median, interquartile range (IQR) and intraclass correlation coefficient (ICC) with 95% confidence intervals (CI). The medians and IQRs refer to the 28 per-examination values, each averaged across the three raters. The ICCs were computed on the item-level ratings of the three raters. The *p*-values of the category-level comparisons and their Holm–Bonferroni adjusted values are reported in the text. The global comparison was the primary endpoint and was not part of this correction.

## Data Availability

The data presented in this study are available on request from the corresponding author. The data are not publicly available due to privacy and ethical restrictions.
